# Efficacy and safety evaluation of citalopram and doxepin on sleep quality in comorbid insomnia and anxiety disorders

**DOI:** 10.3892/etm.2015.2686

**Published:** 2015-08-17

**Authors:** JUNFENG WU, FEI CHANG, HENGBING ZU

**Affiliations:** Department of Neurology, The Jinshan Hospital of Fudan University, Shanghai 201508, P.R. China

**Keywords:** citalopram, doxepin, insomnia, anxiety disorders

## Abstract

Anxiety disorders are frequently comorbid with insomnia, and sleep disturbance in patients with anxiety disorders is the most common complaint. Antidepressants can affect sleep quality; however, their effect in patients with comorbid insomnia and anxiety disorders is unclear. The aim of the present study was to comprehensively evaluate the dose, treatment duration, treatment efficacy and safety of clinical citalopram and doxepin application in patients with comorbid insomnia and anxiety disorders. It was found that both citalopram (20 mg/day) and low-dose doxepin (12.5 mg/day) significantly improved sleep latency, duration and disturbances, as well as daytime dysfunction and the global Pittsburgh Sleep Quality Index during the 12-week treatment period. Notably, low-dose doxepin significantly improved sleep latency in patients after treatment for 8 and 12 weeks as compared with citalopram. It was further observed that both citalopram and low-dose doxepin improved anxiety. A significant and positive correlation was found between the improvement in the sleep quality and anxiety in the two treatment groups. Citalopram and low-dose doxepin both showed good efficacy and a low adverse reaction rate in the treated patients. These data support a potential application of citalopram and low-dose doxepin in the treatment of patients with comorbid insomnia and anxiety disorders.

## Introduction

Anxiety disorders are a group of disorders or syndromes including generalized anxiety disorder (1). The central characteristic of these disorders is persistent, maladaptively triggered anxiety (2). Anxiety disorders most commonly coexist with insomnia, which refers to sleeplessness of any nature (2). Notably, 70–90% patients with anxiety disorders exhibit insomnia (3,4). Compared with normal insomnia in the absence of anxiety disorders, the sleep efficiency and duration and the amount of slow-wave sleep are significantly reduced in comorbid insomnia and anxiety disorders (5). Sleep disturbance in patients with anxiety disorders is a common complaint that can have a profound effect on the course of the anxiety disorder (6); therefore, the study of insomnia that is comorbid with anxiety disorders is particularly important. At present, little is known about the prevention of this type of insomnia.

Current medications for the treatment of insomnia include benzodiazepine-receptor agonists, melatonin and melatonin variants, antidepressants, antipsychotics and antihistamines (7). Among these medications, antidepressants, antipsychotics and antihistamines are usually preferred due to concerns about the tolerance and dependence associated with the other medications (7). Citalopram, as an antidepressant medication, is a selective serotonin reuptake inhibitor (SSRI) (8). It has been noted that citalopram is associated with daytime sedation in patients and could cause an improvement in sleep quality in depressed patients (9). Citalopram results in a decrease in the proportion of rapid eye movement sleep and an increase in the proportion of non-rapid eye movement sleep in depressed patients (10). Doxepin, another antidepressant medication, is a tricyclic drug associated with significant sedative effects in the treatment of insomnia (11). Doxepin has marked inhibitory effects on neurotransmitter receptors, including the histamine, serotonin, α1 adrenergic and muscarinic acetylcholine receptors (12), and has been shown to strongly promote the initiation and maintenance of sleep and to improve sleep quality (11,12). These findings from previous studies suggest the potential effects of citalopram and doxepin on sleep disorders; however, to the best of our knowledge, a comparison between the effects of citalopram and doxepin on sleep quality in patients with comorbid insomnia and anxiety disorders has yet to be conducted. The aim of the present study, therefore, was to compare the effects of citalopram with those of low-dose doxepin on sleep quality in patients with comorbid insomnia and anxiety disorders and to assess the safety of these drugs in clinical application.

## Patients and methods

### 

#### Participants and demographic characteristics

Between September 2009 and January 2013, 78 patients with a diagnosis of anxiety disorders and insomnia, according to the Hamilton Anxiety Rating Scale (HAMA) (score, ≥14) and Pittsburgh Sleep Quality Index (PSQI; score, ≥7) criteria were selected from The Jinshan Hospital of Fudan University (Shanghai, China). The inclusion criteria included patients between 45 and 64 years of age that had not received any psychotropic drugs for ≥2 weeks or hormonal agents and immunomodulators in the 6 months prior to the initiation of the study. The exclusion criteria were severe medical conditions (cancer, cardiovascular and cerebrovascular diseases, thyroid disorders), pregnancy and lactation and mental retardation disorders. All patients provided signed, informed consent. The study was approved by the Ethics Committee of the Faculty of Pharmacy at The Jinshan Hospital of Fudan University.

#### Study design

The study followed a randomized and controlled design. The baseline HAMA and PSQI of each patient were assessed. The patients were then randomly assigned to receive either citalopram (20 mg/day, taken after breakfast) or doxepin (12.5 mg/day, taken 30 min before sleep at night) for 12 weeks (n=39 per group). Sleep quality and the severity of anxiety were measured at weeks 4, 8 and 12 during treatment using the PSQI and HAMA, respectively. Citalopram (Citalop™) was obtained from RPG Life Sciences Ltd. (Mumbai, India), and doxepin (Spectra™) was from obtained from Ranbaxy Ltd. (Gurgaon, India).

#### PSQI assessment

The PSQI is a self-reported questionnaire tool for the subjective assessment of sleep in adults. It measures seven components of sleep, including latency, quality, duration, disturbances, efficiency, the use of sleep medications and daytime dysfunction. For each component, scores can be assigned from 0 to 3 (13). The global PSQI is the sum of the scores for each component. Patients with higher scores in each component or in the global PSQI are more severely affected.

#### HAMA assessment

The HAMA is widely used in clinical and research settings to measure the severity of anxiety symptoms. The scale consists of 14 items defined by a series of symptoms. Each item is scored from 0 (not present) to 4 (severe), with a total score range from 0 to 56. A score of <17 indicates mild severity, 18–24 indicates mild to moderate severity and 25–30 indicates a moderate to severe condition (14).

#### Adverse reactions and missing participants

During drug treatment, the following adverse effects were measured: Headache, aggravated insomnia, blood pressure increase, hyperexcitability, nausea and vomiting, dizziness, palpitations, frequent urination, somnolence and numbness. During the study, 1 patient in the citalopram group and 1 patient in the doxepin group cease to participate due to unknown reasons.

#### Statistical analysis

All statistical analyses were performed using SPSS statistical software, version 17.0 (SPSS, Inc., Chicago, IL, USA). The significance of intra-group comparisons was determined using two-way (treatment × time-course) analysis of variance, while the significance of inter-group comparisons was determined using the Fisher's exact test. P<0.05 was considered to indicate a statistically significant difference. All experiments were repeated ≥3 times, and data are expressed as the mean ± standard error of the mean.

## Results

### 

#### Demographic and clinical characteristics

A total of 78 patients met the inclusion criteria and were randomly assigned to take citalopram or doxepin. Three patients in the citalopram group and 2 patients in the doxepin group did not complete the study due to adverse drug reactions. In total, 35 participants in the citalopram group and 36 participants in the doxepin group completed the study. Two-way, independent samples *t*- and χ^2^ test analyses revealed no significant differences between the two groups with respect to age (P=0.210), gender (P=0.129), initial PSQI (P=0.140) and HAMA (P=0.630). The demographic characteristics of the patients are shown in Table I.

#### Comparison of the effects of citalopram and doxepin on sleep quality

**Figure 1.**
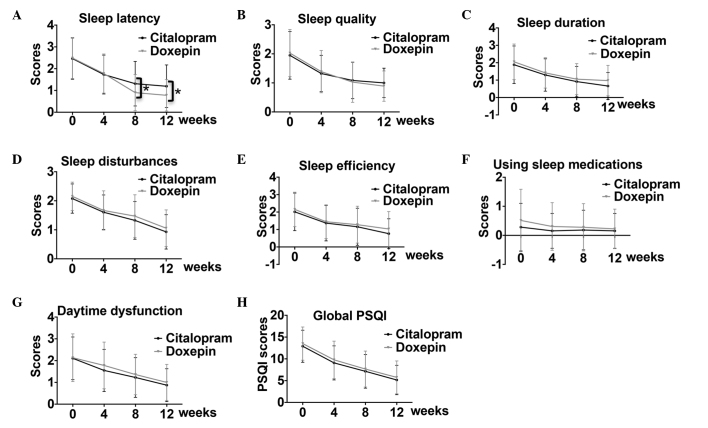
Effects of citalopram and dopexin on the quality of sleep in patients treated for 12 weeks. The (A) sleep latency, (B) sleep quality, (C) sleep duration, (D) sleep disturbances, (E) sleep efficiency, (F) use of sleep medications, (G) daytime dysfunction and (H) global score were measured according to the PSQI. *P<0.05. PSQI, Pittsburgh Sleep Quality Index.

To compare the effects of citalopram and doxepin on sleep quality, the seven components of the PSQI were measured at weeks 0, 4, 8 and 12. The data showed that both citalopram and doxepin treatment gradually improved sleep latency, quality, duration, disturbances and efficiency, as well as daytime dysfunction and global PSQI, but not the use of sleep medications, across the treatment period (Fig. 1). Statistical analysis indicated that, from weeks 0 to 12, citalopram administration led to significant improvements in sleep latency and disturbances, as well as daytime dysfunction and global PSQI (Table II). In addition, citalopram significantly improved sleep quality and duration from weeks 0 to 8 and improved sleep efficiency from weeks 0 to 4 and 8 to 12 (Table II). For patients treated with doxepin, sleep latency, but not daytime dysfunction, were significantly improved from weeks 0 to 12. The sleep quality of patients treated with doxepin showed improvements from weeks 0 to 8. In addition, the sleep disturbance was improved from weeks 0 to 4 and 8 to 12, while sleep efficiency and duration was only improved from weeks 0 to 4 (Table II). Notably, doxepin administration had a significantly greater effect on sleep latency, but not on the other components, at weeks 8 and 12 as compared with citalopram (Fig. 1A). These data suggest that citalopram widely affected the different components of the PSQI over a long-term treatment period as compared with doxepin; however, doxepin had a greater effect on sleep latency than citalopram.

#### Patients treated with citalopram or doxepin exhibit improvements in anxiety

**Figure 2.**
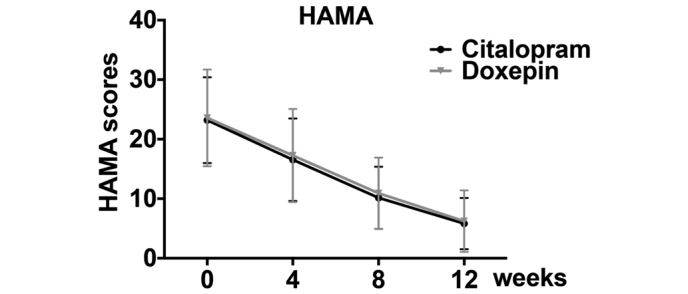
Effects of citalopram and dopexin on anxiety in patients treated for 12 weeks. The severity of anxiety was measured by the HAMA. HAMA, Hamilton Anxiety Rating Scale.

To assess the effects of citalopram and doxepin on the severity of anxiety, the HAMA was used. Both citalopram and doxepin decreased the HAMA scores, suggesting their efficacy in reducing anxiety severity (Fig. 2); however, no significant difference was found between the citalopram and doxepin groups (Table III). These data suggest that citalopram and doxepin are similarly efficacious at decreasing anxiety severity.

#### Positive correlation between sleep quality and anxiety improvement in citalopram- and doxepin-treated patients

**Figure 3.**
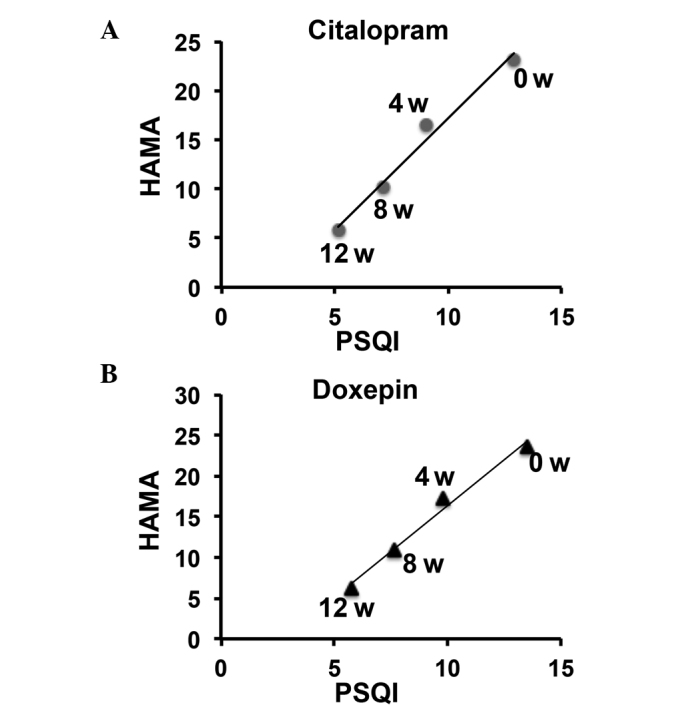
The correlation between improvements in anxiety and sleep quality is significant and positive during (A) citalopram or (B) dopexin treatment. HAMA, Hamilton Anxiety Rating Scale; PSQI, Pittsburgh Sleep Quality Index.

The correlation between sleep quality improvement and anxiety severity in the citalopram and doxepin groups was next examined. A significant and positive correlation between the improvements in sleep quality and anxiety was observed in the citalopram group (P<0.01, r=0.990) and in the doxepin group (P<0.01, r=0.992) (Fig. 3 and Table IV). No significant difference was found between the citalopram and doxepin groups.

#### Efficacy assessment and adverse reactions

The efficacy of citalopram and doxepin treatment was assessed according to the following criteria: i) Rehabilitation (HAMA and PSQI scores improved by >75%); ii) significantly improved (HAMA and PSQI scores improved by >50% but <75%); iii) improved (HAMA and PSQI scores improved by >25% but <50%); and iv) no effects (HAMA and PSQI scores improved by <25%). The efficacy of citalopram and doxepin treatment is presented in Table IV. The data showed that citalopram and doxepin exhibited different efficacies in the treatment of the patients (Table IV). Citalopram promoted rehabilitation in 21.0% of patients and a significant improvement in 60.5% of patients. By contrast, doxepin promoted rehabilitation in 31.6% of patients and a significant improvement in 36.8% of patients. In addition, citalopram and doxepin administration was associated with different adverse drug reaction, as shown in Table V. During this study, 5 patients in the citalopram group and 9 patients in the doxepin group exhibited an adverse reaction, including headache and aggravated insomnia. Overall, citalopram and doxepin showed notable efficacy, with few adverse drug reactions, in the treatment of insomnia in patients with anxiety disorders. Compared with doxepin, citalopram was more effective at inducing a significant improvement and less effective at inducing the rehabilitation of the patients.

## Discussion

Insomnia is a common clinical mental illness that seriously impacts the daily lives and work quality of affected individuals (15,16). Psychiatric disorders, particularly anxiety and mood disorders, have been proposed as a frequent cause of insomniac symptoms (17). Clinical experience shows that the majority of patients with anxiety disorders suffer from sleep disturbances (5,6); however, most of the previous studies that have been conducted focused on primary insomnia, and little investigation into comorbid insomnia and anxiety disorders has been instigated.

Citalopram, a commonly used SSRI, is applied for the clinical treatment of depression and insomnia (10); however, controversy surrounds the role of SSRIs in the treatment of insomnia in patients with anxiety disorders. Several previous studies have shown that SSRI treatment can lead to anxiety and insomnia (18,19). By contrast, another study has suggested that SSRI treatment in patients with insomnia and anxiety disorders causes poorer sleep quality at the beginning but results in greater improvements in sleep time a few weeks later (20). In the present study, the results demonstrated that low-dose citalopram (20 mg/day) markedly improved total sleep duration, sleep quality and daytime dysfunction in patients with anxiety disorders (Fig. 1 and Table II). In addition, citalopram administration resulted in notable improvements in anxiety in the patients (Fig. 2 and Table III). The current data therefore support that the theory that low-dose citalopram used in the treatment of insomnia with anxiety disorders is beneficial.

Doxepin is another antidepressant that is used for the therapy of anxiety and depression. High doses of doxepin cause anti-cholinergic effects and can lead to side effects including increased appetite and weight and decreased blood pressure (11,21). Low-dose doxepin, however, exhibits a more specific effect against H1-receptor antagonists and does not show significant anti-cholinergic effects or result in severe adverse reactions (22,23). In the present study, low-dose doxepin (12.5 mg/day) was used to treat comorbid insomnia with anxiety disorders, and it was found that doxepin significantly improved sleep latency in patients from weeks 0 to 12 (Fig. 1 and Table II). Furthermore, only 2 of the participants (5.3%) exhibited sleepiness as an adverse reaction (Table V). This is consistent with a previous study, in which it was found that low-dose doxepin could improve the sleep quality of insomniac patients (24), thereby supporting a potential application of doxepin in the treatment of comorbid insomnia and anxiety disorders. It has been reported that histamine plays an essential role in the awakening process (25). Considering the specific role of low-dose doxepin against H-receptor antagonists, but not other neurotransmitters, it is reasonable to propose that low-dose doxepin improves sleep quality by delaying the awakening process without affecting appetite and the noradrenaline pathway; however, the detailed mechanisms underlying the action of doxepin require clarification in future studies.

Although the role of citalopram and doxepin in the treatment of insomnia has been investigated in previous studies (10–12,18,21–24,26), the effects of these drugs in patients with comorbid insomnia and anxiety disorders are unclear. In the present study, a comprehensive evaluation of the dose, treatment duration, treatment efficacy and safety of the clinical application of citalopram and doxepin in patients with comorbid insomnia and anxiety disorders was performed. Citalopram and doxepin significantly improved sleep quality during treatment (Fig. 1 and Table II), while doxepin administration resulted in a significantly greater improvement in sleep latency in patients as compared with citalopram (Fig. 1A). In addition, both citalopram and doxepin showed a significant and positive correlation between improvements in sleep quality and anxiety (Fig. 3). The present study therefore suggested that citalopram and doxepin could have a beneficial effect in the therapy of patients with comorbid insomnia and anxiety disorders. Drug resistance, relapse of insomnia, abstinence and drug dependence during treatment were not assessed in the present study; therefore, the possibility that long-term treatment with these drugs could lead to the above effects cannot be excluded. In conclusion, both citalopram and doxepin can improve sleep quality and anxiety in patients with comorbid insomnia and anxiety disorders. Compared with doxepin, citalopram showed a greater efficacy at inducing a significant improvement in patients and less efficacy at inducing rehabilitation during treatment.

## Figures and Tables

**Table I.** Demographic and clinical characteristics of the patients.

Variable	Citalopram, n=39	Doxepin, n=39
Age range (years)	45–64	45–64
Female gender (%)	64.1	79.5
Alcohol- or drug-abuse history	No	No
Psychotropic drug history (in 2 weeks)	No	No
Hormonal agent and immunomodulator history	No	No
PSQI before treatment	≥7	≥7
HAMA before treatment	≥14	≥14

PSQI, Pittsburgh Sleep Quality Index; HAMA, Hamilton Anxiety Rating Scale.

**Table II.** Effects of citalopram and doxepin on PSQI components across time.

PSQI components	Comparison (weeks)	Citalopram (P-value)	Doxepin (P-value)
Sleep latency	0–4	3.62×10^−6^	1.36×10^−3^
	4–8	9.72×10^−3^	1.04×10^−2^
	8–12	9.97×10^−3^	4.37×10^−2^
Sleep quality	0–4	7.05×10^−7^	6.41×10^−4^
	4–8	2.44×10^−2^	3.50×10^−2^
	8–12	3.79×10^−1^	3.42×10^−1^
Sleep duration	0–4	5.27×10^−4^	5.31×10^−3^
	4–8	1.33×10^−2^	8.73×10^−2^
	8–12	6.94×10^−2^	6.91×10^−1^
Sleep disturbance	0–4	5.93×10^−7^	5.84×10^−4^
	4–8	8.02×10^−3^	2.47×10^−1^
	8–12	2.34×10^−4^	1.20×10^−2^
Sleep efficiency	0–4	2.43×10^−4^	2.47×10^−3^
	4–8	2.29×10^−1^	4.88×10^−1^
	8–12	1.61×10^−2^	3.06×10^−1^
Use of sleep medications	0–4	2.86×10^−1^	3.54×10^−1^
	4–8	7.95×10^−1^	8.86×10^−1^
	8–12	7.95×10^−1^	7.54×10^−1^
Daytime dysfunction	0–4	7.14×10^−4^	1.65×10^−1^
	4–8	4.21×10^−2^	8.26×10^−2^
	8–12	1.34×10^−2^	8.64×10^−2^
Global PSQI	0–4	7.07×10^−9^	1.54×10^−4^
	4–8	3.67×10^−3^	3.70×10^−2^
	8–12	1.39×10^−3^	4.37×10^−2^

PSQI, Pittsburgh Sleep Quality Index.

**Table III.** Effects of citalopram and doxepin on the HAMA scores across time.

Index	Comparison (weeks)	Citalopram (P-value)	Doxepin (P-value)
HAMA score	0–4	5.92×10^−8^	1.62×10^−7^
	4–8	5.44×10^−9^	3.82×10^−10^
	8–12	3.01×10^−7^	1.06×10^−8^

HAMA, Hamilton Anxiety Rating Scale.

**Table IV.** Effects of citalopram and doxepin on the sleep quality of patients.

Medications	Rehabilitation, n (%)	Significantly improved, n (%)	Improved, n (%)	No effects, n (%)	Discontinued, n (%)	Total, n
Citalopram	8 (21.0)	23 (60.5)	1 (2.6)	3 (7.9)	3 (7.9)	38
Doxepin	12 (31.6)	14 (36.8)	5 (13.2)	5 (13.2)	2 (5.2)	38

**Table V.** Adverse reactions in citalopram- and doxepin-treated patients.

Medications	Headache (n)	Aggravated insomnia (n)	Increase in BP (n)	Hyperexcitability (n)	Nausea and vomiting (n)	Dizziness (n)	Palpitations (n)	Frequent urination (n)	Somnolence (n)	Numbness (n)
Citalopram	2	2	2	1	0	0	1	1	0	1
Doxepin	3	3	0	0	1	1	1	0	2	0

In total, 5 patients suffered from adverse reactions in the citalopram group and 9 in the doxepin group. BP, blood pressure.
